# Editorial: Meaning in Late-Life

**DOI:** 10.3389/fpsyg.2022.861479

**Published:** 2022-03-10

**Authors:** Jessie Dezutter, Gørill Haugan, Suvi Saarelainen

**Affiliations:** ^1^Faculty of Psychology and Educational Sciences, KU Leuven, Leuven, Belgium; ^2^Department of Public Health and Nursing, Norwegian University of Science and Technology, Trondheim, Norway; ^3^School of Theology, University of Eastern Finland, Kuopio, Finland

**Keywords:** meaning in life, elderly care, meaning making, late life, advanced age, nursing home, sources of meaning in life, presence of meaning in life

The ongoing COVID pandemic showed us how vital the experience of meaning in life is for human wellbeing. This contemporary observation is backed up with cumulating scientific evidence illustrating that a sense of meaning in life relates to more optimal psychological, physical and social functioning as well as to better adjustment to traumatic events (Roepke et al., 2014; Winger et al., 2016; Czekierda et al., 2017; Fischer et al., 2020). We assume that the experience of meaningfulness in life is equally important during the different stages of the life span. Surprisingly, Steger et al. (2009) found that older adults reported even higher levels of meaningfulness compared to younger cohorts. However, in late adulthood, especially among people 80 years and older, chronic illness, loss of functionality, relationships etc. will likely put a strain on the meaningfulness experienced in life: life aspects that were important in generating meaning earlier in life such as care for children, hobbies, travel, or community service might disappear. Meaningful activities like reading, gardening, or sport might become more difficult to perform due to physical decline. Relocation to a nursing home or assistant living unit can weaken the social ties and reduce social contacts, impacting gravely on a crucial source of meaning namely personal relationships (Dewitte et al., 2021). The life stage of advanced age can have the power to erode meaning in the lives of older adults, especially when decay and decline are present.

The articles in this Research Topic provide an overview of how and why experiencing meaning in late life is important for well-being and psychological functioning but also point out the challenges in constructing meaning in old age. The articles are interdisciplinary, coming from nursing studies, psychology, health studies, religious studies & theology, and palliative care. The methodologies implemented are diverse ranging from ethnographic and in-depth interview studies to cross-sectional and longitudinal quantitative studies illustrating how researchers try to address this multilayered and complex phenomenon from different angles and perspectives. The empirical studies in this special issue are complemented by theoretical analysis articles and scoping reviews.

The ***phenomenon*** of perceived meaning in life calls for further study. At this moment, most researchers adhere to the tripartite view on meaning in life (George and Park, 2016; Martela and Steger, 2016), making a distinction between “purpose,” “mattering,” and “coherence” as essential dimensions of the experience of meaning. However, the discussion on which dimensions do belong to the core of the phenomenon and which do not is still unsettled. In this Research Topic, Pachana and Baumeister propose six potential dimensions of meaning in life based on earlier models and theorizing. They summarize purpose, value, efficacy, self-worth, mattering, and comprehension as central aspects of perceived meaning in life. Knizek et al. took another approach and zoomed in on how meaning in life is experienced by extremely poor Ugandan older adults. Here, respect was a crucial facet concretized in the ability and willingness of possible descendants to support their aged parents. Covering of basic needs and receiving dignities were needed to perceive life as meaningful despite the extreme poverty. Possibly, these findings of Knizek associate with the dimensions of self-worth and mattering that are put forward by Pachana and Baumeister.

An interesting alley is the focus on the ***sources*** that provide meaningfulness in life. Earlier research (Dewitte et al., 2021) showed that not all sources of meaning are equally related to the experience of meaning. Sørensen et al. came to the same conclusion based on a large register of Norwegian older adults. In their study, vertical self-transcendence, including explicit religiosity and spirituality, had the strongest relation to meaningfulness for people in late adulthood. Moreover, earlier accomplishments, including generativity and unselfish engagement with the surroundings and future generations, were more strongly related to the experience of meaningfulness in comparison with other life aspects. Tiilikainen et al. addressed the question of how meaningfulness is experienced by older adults during the COVID-19 pandemic: their mixed-method study reveals that social contacts, daily chores and activities, familiar places and seasonal changes contribute to meaningfulness during the pandemic situation. Still, studies on sources of meaning in life are predominantly cross-sectional and Sorensen therefore rightly calls for longitudinal research and interventional studies to clarify more precisely how sources of meaning are related to a sense of meaningfulness. In addition to longitudinal studies, we also call for studies with an experimental sampling methodology in order to elucidate the finer daily dynamics between sources and experience. Slettebø et al. assessed the sources that contribute to ***daily meaning*** for residents in nursing homes, revealing five themes: (1) opening the nursing home to the surroundings; (2) expanding and strengthening tis he community of practice; (3) facilitating customized activities; (4) ensuring sufficient nutrition and facilitating enjoyable mealtimes; and (5) preventing unrest and disturbing behavior. Despite some similarities (engagement with others, social contacts, community) in the findings of these three studies on resources of meaning in late life (Sørensen et al.; Tiilikainen et al.) the identified sources represent a large diversity. The variety in study population and research design can possibly explain this diversity. Nevertheless, these diverse findings do trigger follow-up questions regarding the inter-individuality or situation-specificity of meaning sources in their contribution to a sense of meaning.

In line with the general literature on meaning in life, several articles in this Special Issue demonstrate associations between meaningfulness and aspects of ***psychological functioning*** ***in late life***. Lewis and Hill scrutinized “purpose” as a dimension of meaning in life showing that a sense of purpose in life might buffer the negative association between depressive symptomatology and cognitive functioning in older adults. This study indicates that sense of purpose can act as a resilience factor and thus adds to a growing body of research suggesting that a sense of purpose contributes to cognitive reserve. Araújo et al. showed that experiencing meaningfulness in life is a vital factor associated with being willing to live longer in centenarians. Further, the Special Issue contributes on knowledge how loss or lack of meaning is linked with difficulties in multiple aspects of life. Golovchanova et al. showed significant associations between perceived meaning and health problems, psychosocial functioning, and social support among community-residing older adults in Sweden. Appel et al. illustrated how a lack of meaning in life is often a central aspect in the experiences of tiredness or weariness of life in older adults. They provide a scoping review that clearly points out the lack of conceptual consensus in this field, as well as the complexity of the tiredness-of-life phenomenon and how existential concerns like meaning in life are probably intertwined with the tiredness-of-life experience.

A vital question concerns how meaning is constructed or made: which underlying mental processes are important in the ***meaning-construction processes***? Dewitte and Dezutter focused on meaning reflectivity as a potential process in meaning-generation. They showed that community-residing older adults who are indifferent about issues of meaning in life might be more vulnerable to experiencing meaninglessness and depressive symptoms. Other scholars have investigated specific types of meaning-making processes. For example, Spännäri and Hanne Laceulle studied meaning making among Finnish retirement migrants; a key finding was that meaning making occurs as a process that is often inherent to daily activities which may seem “trivial,” but in fact turn out to be important sources of purpose, values, and connectedness. Meaning seems to be constructed in the details of life. Moreover, religion and spirituality seems to be intertwined with meaning making processes. Nissen et al. showed that spiritual care involves meaning making processes. Their theoretical analysis implies that meaning-related concerns of older patients can be addressed as a part of spiritual care. Along these lines, Toussaint et al. investigated the role of forgiving as a potential meaning making process in adapting to the COVID-19 pandemic situation. These findings not only show that older age relates to better mental health through higher levels of meaningfulness for but also that older age relates to better mental health through a serial indirect pathway. Lower COVID-19-related stress perceptions were associated with higher presence of meaning, higher forgiveness and better mental health. Positive psychological processes might thus play a pivotal role in meaning making. Meaning making can also be a difficult task. Nilsen et al. showed how crucial but challenging this process can be for breast-cancer survivors.

Meaning in life is fundamental to well-being and life quality. Within the care for older adults, the way that health care professionals (HCPs) address meaning in life-concerns is extremely relevant for shaping individualized and person-oriented care that goes further than the pure biomedical aspects of illness and suffering. In a qualitative study Isene et al. showed that HCPs in dementia care actively search for that what is meaningful for adults with dementia. The authors state that professional attentiveness for the patients' expressions and experiences of meaning may contribute to improved dementia care. HCPs, however, do not automatically have a focus on meaning in life as an essential part of care, as is described in Isene's study. Accordingly, an increased attention for existential topics in elderly care is advocated. This is in line with the findings of Viftrup et al. who showed in a qualitative study with hospice patients that meaning in life and other existential concerns are not easily talked about. Hospice patients tend to use medical solution-focused vocabulary when they talk about their meaning at the end of their lives. Dialogues with HCPs covering existential topics seem scarce and patients' existential vocabulary seems limited. The findings presented by Isene et al. and Vifturp et al. call for more attention for meaning-related aspects in health care practice. The study of Wynn et al. offers a caveat on this topic. Their results show that both care recipients and their partners report a decline in sense of purpose after being diagnosed with dementia; however, their perception of how the other partner evaluates the meaning in life is not the same. The partner of the person with dementia tend to underestimate the sense of purpose felt by the care recipient pointing out that people with dementia still report better well-being than negative stereotypes may suggest. This potential bias is of course an important focal point in dementia care.

In sum, this Research Topic offers insight in the complex phenomenon of meaning in life, its potential sources, the underlying processes, and its correlates. All contributions, however, also identify gaps and lacks in knowledge in this field. More needs to be done before we can fully understand the intriguing and multilayered experience of meaning in life in old age.

## Author Contributions

JD drafted this contribution. JD, GH, and SS revised this contribution. All authors contributed to the article and approved the submitted version.

## Funding

This article is partly funded by the Constructive Advanced Thinking Grant of the Network of European Institutes for Advanced Study. The grant was received by JD, GH, and SS.

## Conflict of Interest

The authors declare that the research was conducted in the absence of any commercial or financial relationships that could be construed as a potential conflict of interest.

## Publisher's Note

All claims expressed in this article are solely those of the authors and do not necessarily represent those of their affiliated organizations, or those of the publisher, the editors and the reviewers. Any product that may be evaluated in this article, or claim that may be made by its manufacturer, is not guaranteed or endorsed by the publisher.
